# Supplementary material for: Combination of Recombinant Proteins S1/N and RBD/N as Potential Vaccine Candidates
Source: Vaccines (Basel). 2023 Apr 18;11(4):864. doi: 10.3390/vaccines11040864 (PMC10142685; doi:10.3390/vaccines11040864)
Supplement: Supplementary file 1 [file vaccines-11-00864-s001.zip › vaccines-2270112-supplementary.pdf]

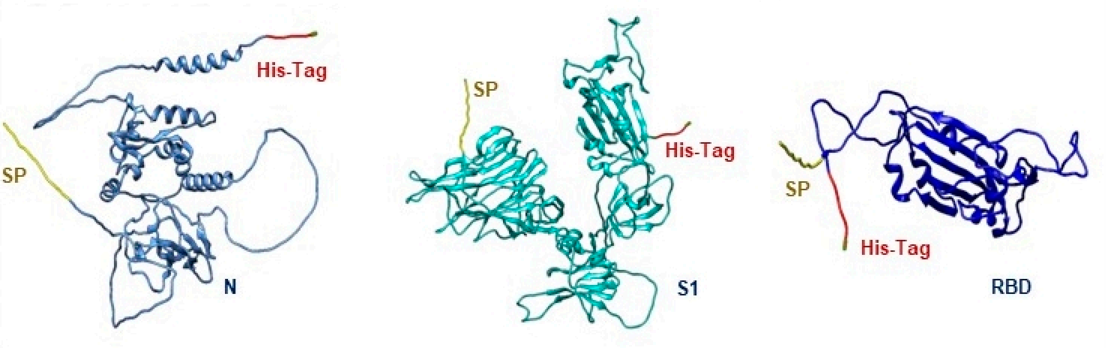

(D)

SeqName	Position	Potential	Jury agreement	N-Glyc result
Sequence	61 NNTA	0.6753	(9/9)	++
Sequence	91 NSSP	0.2119	(9/9)	---
Sequence	206 NSSR	0.4478	(6/9)	-
Sequence	210 NSTP	0.1286	(9/9)	---
Sequence	283 NVTQ	0.8208	(9/9)	+++

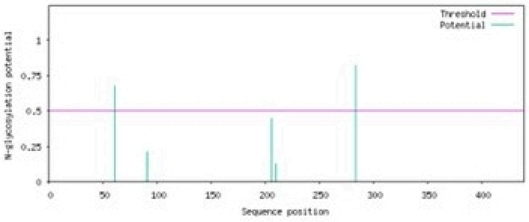

(E)

SeqName	Position	Potential	Jury agreement	N-Glyc result
Sequence	17 NLTT	0.6704	(8/9)	+
Sequence	61 NVTW	0.7860	(9/9)	+++
Sequence	74 NGTK	0.7239	(9/9)	++
Sequence	122 NATN	0.6798	(8/9)	+
Sequence	149 NKSW	0.6300	(7/9)	+
Sequence	165 NCTF	0.6180	(8/9)	+
Sequence	234 NITR	0.7560	(9/9)	+++
Sequence	282 NGTI	0.7290	(9/9)	++
Sequence	331 NITN	0.5783	(7/9)	+
Sequence	343 NATR	0.5446	(7/9)	+
Sequence	603 NTSN	0.5312	(5/9)	+
Sequence	616 NCTE	0.6748	(9/9)	++
Sequence	657 NNSY	0.4208	(7/9)	-
Sequence	709 NNSI	0.3027	(9/9)	---
Sequence	717 NFTI	0.5847	(9/9)	++

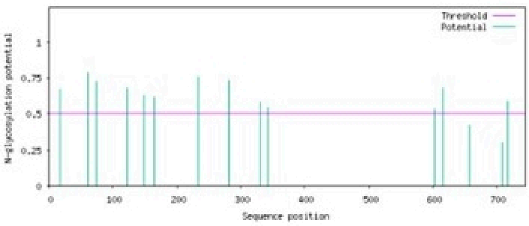

(F)

SeqName	Position	Potential	Jury agreement	N-Glyc result
Sequence	27 NITN	0.6459	(8/9)	+
Sequence	39 NATR	0.6139	(8/9)	+

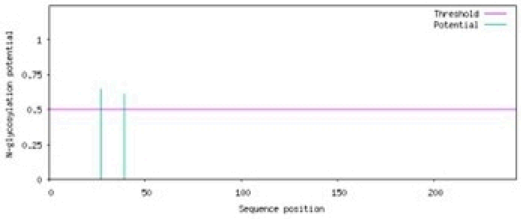

Figure S1: Model of 3D Structure of N (A) S1 (B) and RBD (C) proteins was predicted using Alphafold server. Signal peptide is colored in yellow, and His tag is colored in red. (E-F) Glycosylation sites were predicted in NetNGlyc 1.0 server. Mayor values of 0.5 was considered as positive. Then we analyze by using the server UCLA-DOE LAB), we found the next Overall Quality Factors, S1=77.21,N=80.83 and RBD= 72.77
